# Opportunities for transitional care and care continuity following hospital discharge of older people in three Nordic cities: A comparative study

**DOI:** 10.1177/14034948221122386

**Published:** 2022-09-15

**Authors:** Ann E.M. Liljas, Jutta Pulkki, Natasja K. Jensen, Esa Jämsen, Bo Burström, Ingelise Andersen, Ilmo Keskimäki, Janne Agerholm

**Affiliations:** 1 Department of Global Public Health, Karolinska Institutet, Sweden; 2 Faculty of Social Sciences, Tampere University, Finland; 3 Department of Public Health, Copenhagen University, Denmark; 4 Gerontology Research Centre (GEREC), Tampere, Finland; 5 Faculty of Medicine and Health Technology, Tampere University, Finland; 6 Centre of Geriatrics, Tampere University Hospital, Finland; 7 Finnish Institute for Health and Welfare, Helsinki, Finland

**Keywords:** Ageing, care continuity, care transitions, care systems, hospital discharge, older adults, health services research

## Abstract

**Aim:**

To outline and discuss care transitions and care continuity following hospital discharge of older people with complex care needs in three Nordic cities: Copenhagen, Tampere and Stockholm.

**Methods:**

Data on potential pathways following hospital discharge of older people were obtained from existing literature and expert consultations. The pathways for each system were outlined and presented in three figures. The hospital discharge process of the systems was then compared.

**Results:**

In all three care systems, the main care path from hospital is to home. Short-term intermediate healthcare can be provided in all three systems, possibly creating additional care transitions; however, once home, extensive home healthcare may prevent further care transitions. Opportunities for continuity of care include needs assessments (all cities) and meetings with the patient about care upon return home (Copenhagen, Stockholm). Yet this is challenged by lack of transfer of information (Tampere) and patients’ having to apply for some services themselves (Tampere, Stockholm).

**Conclusions:**

**Comparisons of the discharge processes studied suggest that despite individual care planning and short- and long-term care options, transitional care and care continuity are challenged by limited access as some services need to be applied for by the older person themselves.**

## Background

Advanced age is associated with comorbidity and complex care needs, increasing the pressure on the care sectors [1]. Hospital discharge of older people with complex care needs is challenging as it involves multiple activities and care teams [2]. Effective cooperation between hospital, primary care and home care can reduce the risk of adverse health events, including hospital readmission [3] and unnecessary emergency department visits [4].

To provide high-quality care that meets the complex care needs of the growing ageing populations, care systems may benefit from focusing more on transitional care and care continuity [5–7]. Transitional care originates in discharge management [8] and seeks to ensure continuity of healthcare as patients transfer between and within sites [9]. The aim of this study was to outline and compare care transitions and care continuity following hospital discharge of older people with complex care needs in three Nordic cities: Copenhagen (Denmark), Tampere (Finland) and Stockholm (Sweden). Greater Copenhagen (1.35 million inhabitants across 17 municipalities) has eight hospitals with emergency services, of which four are open 24-7. Tampere is Finland’s third most-populous municipality and encompasses the Pirkanmaa Hospital District, which offers emergency care 24-7 at the hospital and organises secondary and tertiary healthcare to over 500,000 residents across 23 municipalities. In Greater Stockholm (2.2 million residents across 26 municipalities) there are 12 hospitals with emergency services, of which seven are open 24-7.

Comparing the hospital discharge pathways of three Nordic cities is of interest as their populations are among the world’s oldest and located in relatively comparable welfare states with decentralised, tax-funded healthcare and home care systems. It is further important to understand the underlying constructs of these care systems when analysing and comparing Nordic care data. In specific, this study will help in forming the knowledge base of the extended research project Social Inequalities in Ageing (SIA) (sia-project.se), which this study is part of.

## Methods

Data on possible pathways following hospital discharge of older people were collated from literature and consultations with experts known to the research team. The mapping exercise of outlining potential pathways for each system was undertaken by researchers NJ (Copenhagen), JP (Tampere) and AL (Stockholm) with input from the co-authors, and presented as figures. The figures were verified by the experts consulted.

## Results

**Figure 1.**
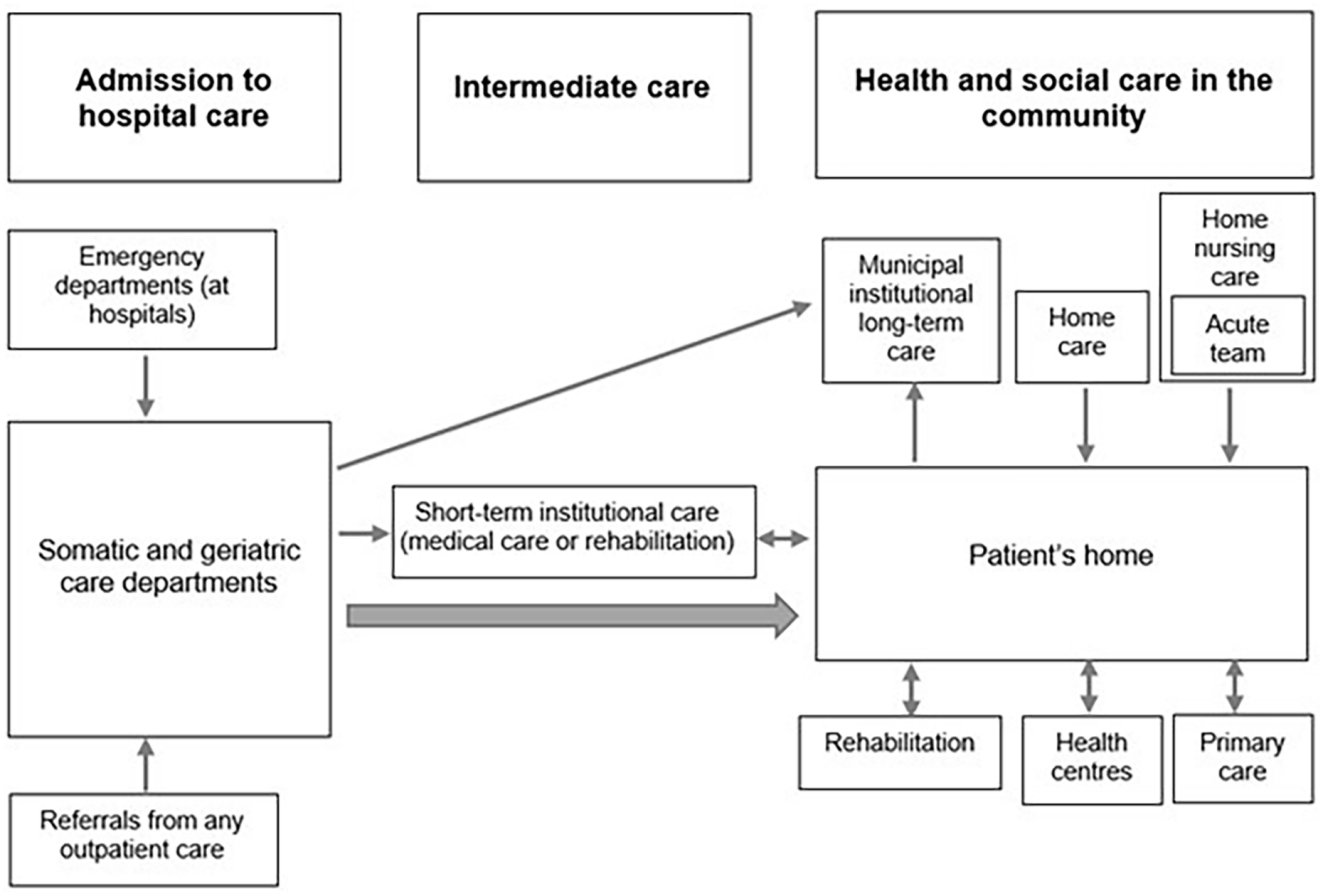
Transitions from hospital to community in Copenhagen Municipality.

In all three cities, following a hospital stay, most older people return home (with/without home healthcare and/or home help). Figure 1 shows care pathways from hospital to community for older people in Copenhagen. Besides returning home, some may be transferred to intermediate care in the form of short-term institutional care or long-term nursing home. Short-term care is provided if there is a need for complex nursing care or rehabilitation. Prior to discharge, the need for home healthcare and home help is assessed at the hospital. The municipality, responsible for both home healthcare and home help, conducts an individual assessment of the patient’s needs and arranges for necessary initiatives such as home visits. Patients in need of extensive support will be assigned a coordinator who organises the treatment and communication between the patient, relatives and the hospital [10]. Home healthcare involves visits to a municipal health centre, home visits by a nurse and, more recently, home visits by the acute team – a municipal home nursing care service that delivers specialised care and treatment [11].

**Figure 2.**
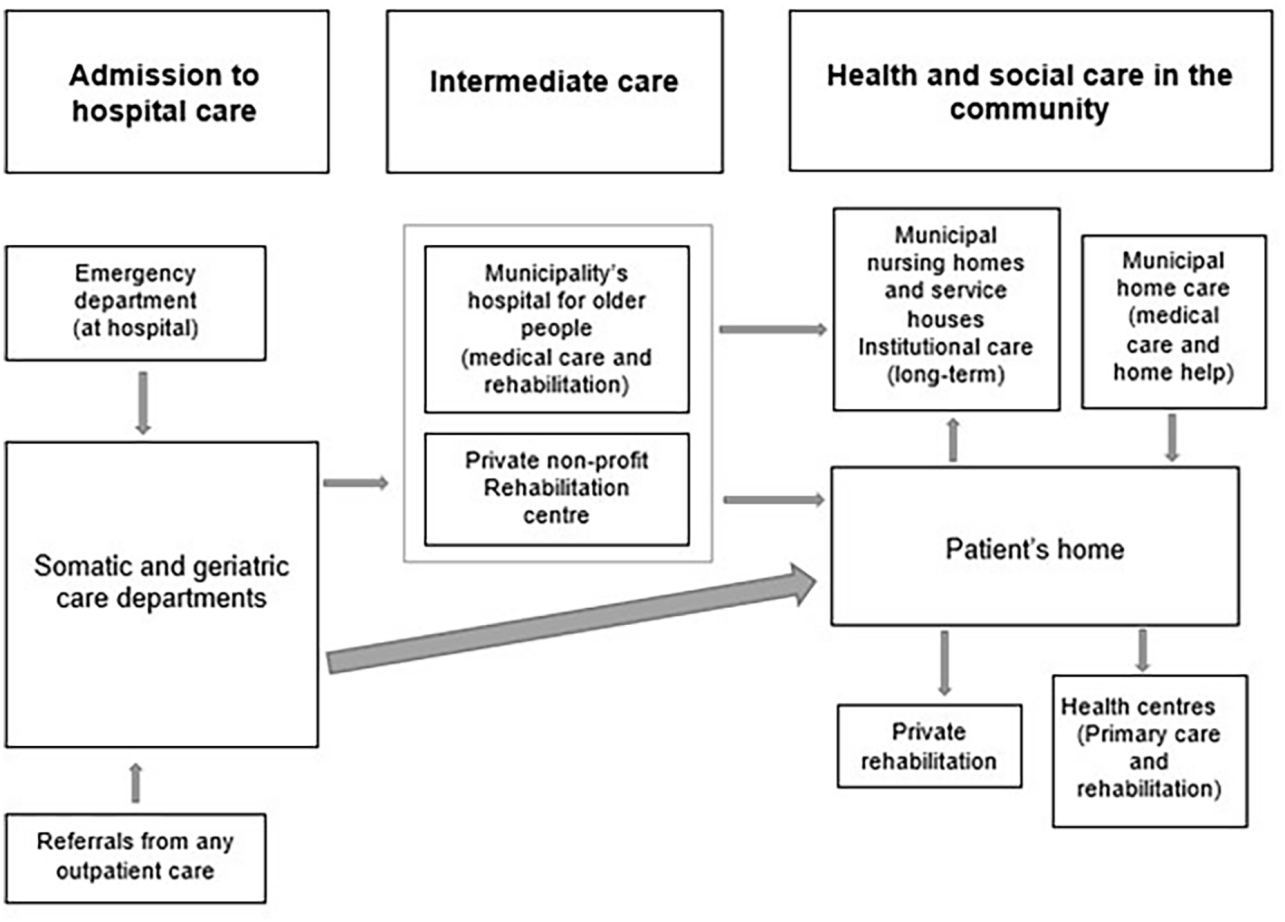
Transitions from hospitals to community in Tampere Municipality.

Figure 2 shows pathways following hospital discharge for older people in Tampere. Depending on the older patient’s needs, home healthcare requirements are assessed (existing home healthcare needs may be reassessed) by the municipality’s home healthcare team immediately after discharge. Hospital staff assist with contacting the home healthcare staff with the older patient’s permission. Patients who have not received home healthcare prior to the hospitalisation are responsible for transferring information between secondary (hospital) and primary healthcare (health centres) themselves [12]. Intermediate care is offered to those in need of intensive medical attention or rehabilitation, who then return home, to a service house or to a nursing home (prioritised to very frail older people). Gaining a place at a service house or nursing home requires a permanent long-term care decision from the municipality, based on care needs assessments conducted while receiving intermediate care or at home [12].

**Figure 3.**
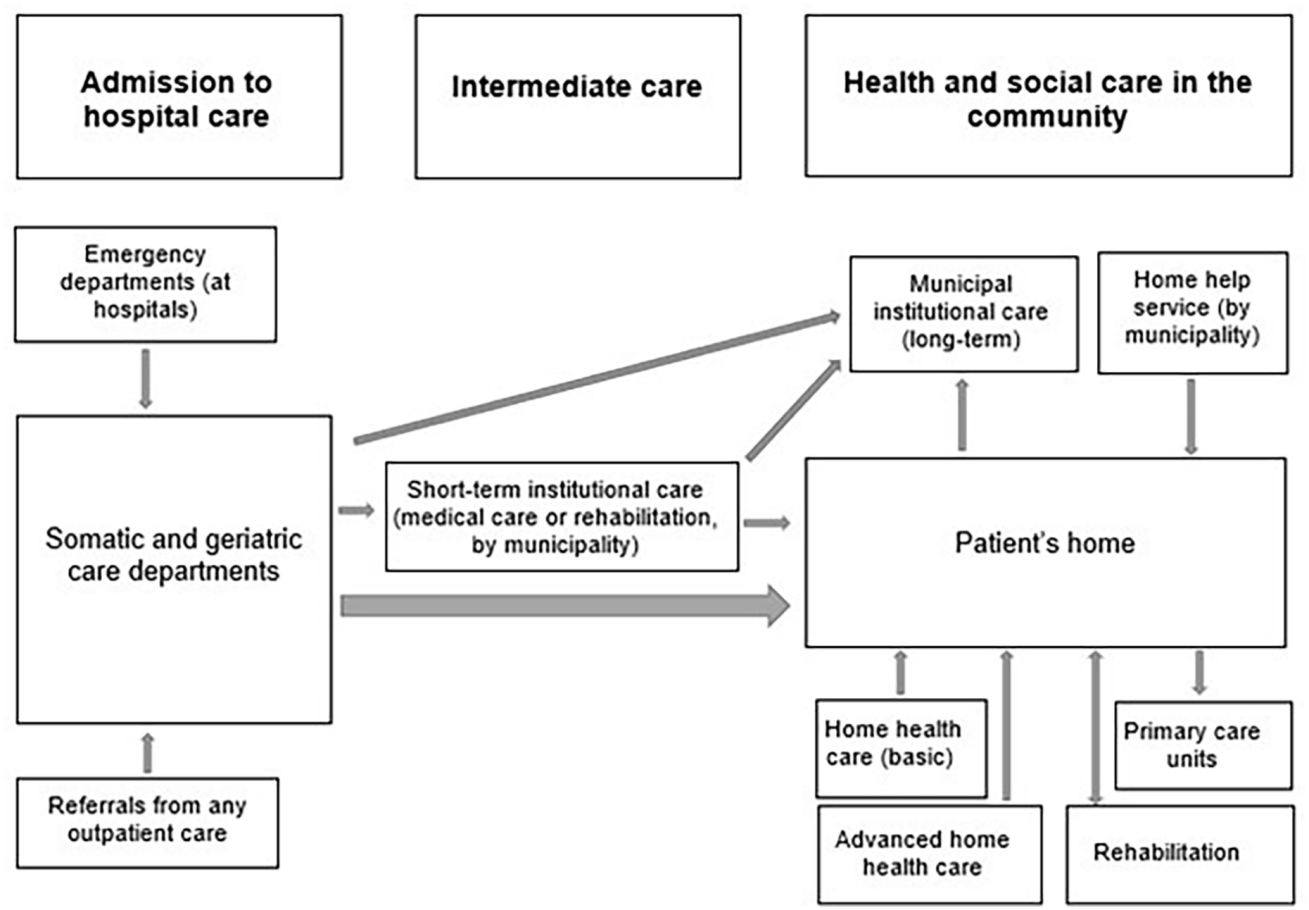
Transitions from hospital to community in Region Stockholm.

In Stockholm, following a hospital stay, short-term care can be provided to patients with difficulties that severely affect their ability to return home (Figure 3). Once back home, a nurse from the patient’s general practice, who will deliver any future home healthcare needed, arranges for an initial care planning meeting. The municipality, responsible for providing home help, is invited if the nurse or patient considers home help relevant. However, the older person will have to apply for home help services themselves. Further, if needed, hospital staff will provide advanced home healthcare. Rehabilitation can be provided in the older person’s home by a provider of their choice. If the older person is very frail, long-term institutional care may be offered at the care planning meeting [13].

## Discussion

All three systems offer intermediate care; through short-term institutions (Copenhagen, Stockholm) and the municipality’s hospital or rehabilitation centre (Tampere). Whilst intermediate care may increase the number of care transitions, the extensive and timely provision of home healthcare and home help in the three systems has the potential to prevent further care transitions. Opportunities for care continuity include needs assessment (all cities) and individual care planning meetings upon return home (Copenhagen, Stockholm). Yet this is challenged by lack of transfer of information (Tampere) and the necessity for patients to apply for some services themselves (Tampere, Stockholm). For instance, in Tampere, patients without home care (healthcare and home help) will receive healthcare from the health centres only if they apply for it themselves. Subsequently, care continuity is only secured for current home care clients and long-term residents. Further, contrary to Copenhagen and Tampere, home healthcare and home help are separately organised and provided in Stockholm: the primary care nurse will arrange for home healthcare only; individuals in need of home help too will have to apply for this themselves. This application process is facilitated by the municipal social care manager if they attend the individual care planning meeting. However, if the older person needs only home help and no home healthcare, it is the individual’s responsibility to contact the municipality. Limited access to some services does not only challenge care continuity, it also indicates system failure [14]. Fragmented care systems are strongly linked to poor care continuity [15], yet little is known about care continuity across healthcare and home care systems. Further research should explore how issues related to care continuity and transitional care in the Nordic care systems could be addressed.

The focus on care transitions and continuity on service system levels provides perspectives of hospital discharge less studied [16]. Limitations include that the study is descriptive and shows only how the systems are structured, not how they work in practice. Further, the findings do not cover important aspects of costs and are restricted to three specific care systems, making it difficult to generalise the findings. Nevertheless, the comparison could facilitate the planning of structural changes and future policy.

## Conclusions

Comparisons of the discharge processes studied suggest that despite individual care planning and short- and long-term care options, transitional care and care continuity are challenged by limited access as some services need to be applied for by the older person themselves.
